# FedPome: federated deep learning for real-time pomegranate disease classification

**DOI:** 10.3389/fpls.2026.1906269

**Published:** 2026-08-27

**Authors:** Lokesh S, Akshaya Prathiksha C. K., Aishwaryalakshmi M., Vishalini V., Yenugonda Hari Krishna, Aparna Mohanty

**Affiliations:** 1 School of Computer Science Engineering and Information Systems, Vellore Institute of Technology, Vellore, India; 2 School of Electronics Engineering, Vellore Institute of Technology, Vellore, India

**Keywords:** deep learning, federated learning, MobileViT, ONNX, pomegranate disease classification, precision agriculture, ViT

## Abstract

Pomegranate cultivation faces significant productivity losses due to fungal and bacterial diseases, yet existing automated detection systems rely on centralized deep learning pipelines that require raw image data aggregation, raising data privacy concerns in distributed agricultural settings. This paper proposes a federated learning (FL) framework for raw-data-decentralized pomegranate disease classification, systematically evaluating six architecturally diverse deep learning models such as Custom CNN, ResNet50, ConvNeXt_V2, ViT-B/16, EfficientNetV2-S, and MobileViT-S (under the FedAvg aggregation protocol across five simulated clients and 10 communication rounds). All six architectures are initialized with publicly available ImageNetpretrained weights to ensure a fair architectural comparison. Experiments are conducted on a working set of 6,823 images derived from the Mendeley Pomegranate Fruit Diseases Dataset (5,099 original images) spanning five disease classes—*Alternaria*, anthracnose, bacterial blight, *Cercospora* fruit spot, and healthy—using a split-before-augmentation pipeline: the train/validation/test split is performed prior to any augmentation; augmentation is applied only to the training partition (5,800 images), while the validation (511) and test (512) sets contain only original, unaugmented images. EfficientNetV2-S achieves the single highest test accuracy of 99.22%, with ViT-B/16, ResNet50, and ConvNeXt_V2 tied at 98.83% and MobileViT-S close behind at 98.63%, offering the strongest efficiency–accuracy tradeoff. We further report results under non-IID (Dirichlet-partitioned) client distributions, centralized and local-only training baselines, and multi-seed statistical evaluation. All six models are exported to ONNX format and deployed as a real-time web application on Hugging Face Spaces, demonstrating practical deployment readiness. The results confirm the viability of federated learning for high-accuracy, raw-data-decentralized crop disease classification while also characterizing its performance degradation under realistic non-IID conditions.

## 1 Introduction

Pomegranate (*Punica granatum*) is a commercially significant crop cultivated across South Asia, the Middle East, and the Mediterranean, yet its yield is severely threatened by fungal and bacterial diseases including *Alternaria* fruit rot, anthracnose, bacterial blight, and *Cercospora* fruit spot. Early and accurate disease identification is critical to minimizing crop loss, but traditional manual inspection is time-consuming, requires domain expertise, and is impractical at scale.

Deep-learning-based image classification has demonstrated strong potential for automated plant disease detection, with centralized approaches dominating existing work (Bhange and Hingoliwala, 2015; Mohanty et al., 2016; Ramcharan et al., 2017; Bhange and Gavali, 2023; Kumar and Sharma, 2023; Mohammed et al., 2025; Pakruddin and Hemavathy, 2025; Revathi and Agash, 2025; Sandhi et al., 2025). However, existing approaches for pomegranate disease diagnosis predominantly adopt centralized training paradigms that aggregate raw farm imagery on a single server (Bhange and Hingoliwala, 2015; Bhange and Gavali, 2023; Kumar and Sharma, 2023; Mohammed et al., 2025; Pakruddin and Hemavathy, 2025; Revathi and Agash, 2025; Sandhi et al., 2025). This raises data privacy concerns in real-world deployments where farm-level image data may be proprietary or geographically distributed across multiple owners.

Federated learning (FL) (Li et al., 2020) addresses these concerns by enabling collaborative model training across decentralized clients without sharing raw data. Despite its promise, no prior study has benchmarked multiple heterogeneous deep learning architectures within a unified FL framework for pomegranate disease classification nor incorporated ONNX-based cross-platform deployment.

This paper addresses these gaps with four key contributions:

1. A federated training framework with FedAvg (McMahan et al., 2017) across five clients and 10 rounds,
2. Systematic benchmarking of six architectures spanning CNNs, transformers, and hybrid models,
3. A comprehensive accuracy–efficiency trade-off analysis including ONNX model size and CPU inference latency, and
4. A publicly accessible real-time disease classification web application deployed via Docker and Hugging Face Spaces at https://lokes123-pomdetect.hf.space.

## 2 Related work

Automated pomegranate disease detection has advanced considerably over the past decade. Early work demonstrated the feasibility of classical image segmentation and feature extraction for detecting common fungal and bacterial diseases from field-captured fruit images in smart-farming contexts (Bhange and Hingoliwala, 2015). The adoption of deep neural networks significantly raised classification performance: a hybrid model combining CNNs with the Honey Badger Optimization Algorithm reduced misclassification errors through meta-heuristic feature selection (Bhange and Gavali, 2023), while a hybrid optimal attention capsule network used spatial attention mechanisms to focus on discriminative lesion regions and suppress background noise (Kumar and Sharma, 2023). More recently, a nature-inspired deep learning optimization framework leveraged evolutionary algorithms to identify near-optimal hyperparameter configurations across multiple architectures for pomegranate disease detection (Sandhi et al., 2025). The most advanced single-model contribution combines deep learning diagnosis with a Retrieval-Augmented Generation (RAG) large language model, enabling not only disease classification but also severity estimation and personalized treatment recommendations (Revathi and Agash, 2025).

On the benchmarking and dataset front, a systematic comparison of CNN, VGG16, ResNet, and MobileNet on the Mendeley pomegranate disease dataset—the same benchmark used in this study— established a direct performance baseline for centralized approaches (Pakruddin and Hemavathy, 2025). A new geographically diverse dataset collected from farms in Halabja, Iraq, was also published to facilitate research on model generalization across different cultivation environments (Mohammed et al., 2025). Beyond pomegranate-specific work, deep learning has demonstrated broad applicability in plant disease detection (Mohanty et al., 2016; Ramcharan et al., 2017), with vision-based methods achieving high accuracy on large-scale crop disease benchmarks such as PlantVillage.

Federated learning (FL) enables collaborative model training across decentralized clients without sharing raw data. A comprehensive survey identified non-IID data distributions, communication overhead, and system heterogeneity as the primary challenges and established FedAvg (McMahan et al., 2017) as the dominant aggregation algorithm (Li et al., 2020). FL has been applied to privacy-preserving plant disease classification, demonstrating its viability for agricultural imaging tasks under data privacy constraints. However, all prior studies on pomegranate disease classification employ centralized training pipelines (Bhange and Hingoliwala, 2015; Bhange and Gavali, 2023; Kumar and Sharma, 2023; Mohammed et al., 2025; Pakruddin and Hemavathy, 2025; Revathi and Agash, 2025; Sandhi et al., 2025), and no existing work systematically benchmarks heterogeneous architectures—CNNs, residual networks, vision transformers, and hybrid models—within a unified FL framework for this crop nor incorporates ONNX-based cross-platform deployment.

## 3 Methodology

**Figure 1:**
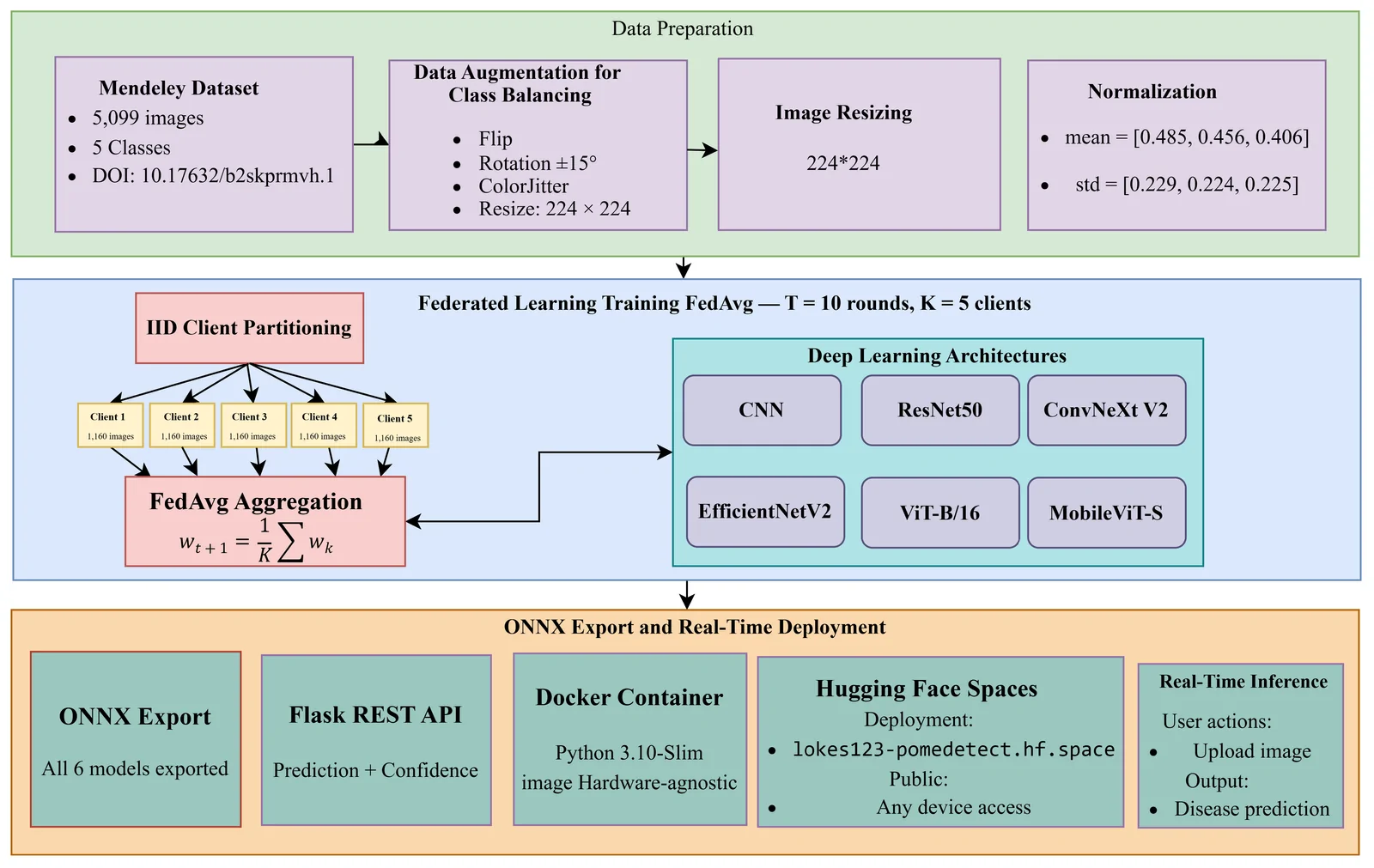
Proposed FL-based pomegranate disease classification framework.

This study proposes a federated deep learning framework for automated pomegranate disease classification using high-resolution fruit images. The pipeline integrates dataset preparation, image preprocessing, class balancing, and multi-architecture deep learning training under a federated averaging (FedAvg) protocol (McMahan et al., 2017) and ONNX-based real-time deployment. The framework aims to achieve high classification accuracy while keeping raw image data decentralized (raw-data-decentralized) across distributed agricultural nodes, without raw image sharing, though we do not claim formal privacy guarantees such as differential privacy or secure aggregation (see “Discussion”). The overall framework architecture is illustrated in **Figure 1**.

### 3.1 Dataset description

**Figure 2:**
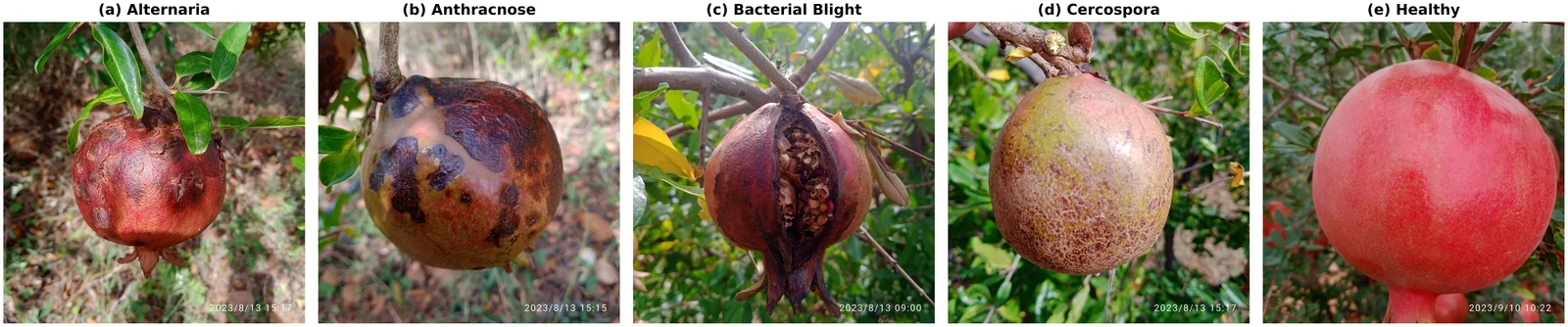
Representative sample images for each of the five disease classes: **(a)** *Alternaria*, **(b)** anthracnose, **(c)** bacterial blight, **(d)** *Cercospora*, and **(e)** healthy. Images sourced from Pakruddin, B., and Hemavathy, R., Pomegranate fruit diseases dataset for deep learning models, Mendeley Data, V1, 2023. DOI: 10.17632/b6s2rkpmvh.1. Licensed under CC BY 4.0.

Experiments were conducted on the Pomegranate Fruit Diseases Dataset (Pakruddin and Hemavathy, 2023), publicly available on Mendeley Data (DOI: 10.17632/b6s2rkpmvh.1, CC BY 4.0). The dataset comprises 5,099 high-resolution images (≈3,120 × 3,120 pixels) of pomegranate fruits across five categories: *Alternaria*, anthracnose, bacterial blight, *Cercospora* fruit spot, and healthy. Images were collected under natural field conditions from pomegranate farms in Ballari, Bengaluru, and Bagalakote, Karnataka, India, capturing real-world variations in illumination, background, and fruit orientation. **Figure 2** shows a representative sample image for each of the five disease classes.

### 3.2 Data preprocessing

All samples were resized to 224 × 224 pixels using GPU-accelerated batch resizing with bilinear interpolation and antialiasing. Pixel intensities were normalized using ImageNet statistics (mean = [0.485,0.456,0.406], std = [0.229,0.224,0.225]) to standardize input distributions across all models.

### 3.3 Dataset balancing and augmentation

The original dataset exhibits class imbalance. The 5,099 original images are first partitioned (seed = 42) into training (80%), validation (10%), and test (10%) subsets on a per-class, per-original-image basis. Augmentation is then applied exclusively to the training partition, using random horizontal flip, vertical flip, rotation (± 15^°^), and color jitter (brightness/contrast ±0.3, saturation ±0.2), with each augmented image traceable to its training-split source image only. After augmentation, the training set is balanced to 1,160 images per class (5,800 training images total). The validation and test sets are left unaugmented and therefore retain the natural (imbalanced) class distribution of the source dataset (511 validation and 512 test images in total), which we consider more representative of real-world disease prevalence. Because the validation and test sets are then unequal across classes, we report macro-averaged precision, recall, and F1-score throughout, which are robust to this imbalance. We additionally verified the splits using a GPU-accelerated perceptual-hash (dHash) near-duplicate check (Hamming distance ≤ 5) between the training set and each of the validation and test sets; this identified 16 near-duplicate pairs out of approximately 6,823 images (*<*0.25%), attributable to consecutive field photographs of the same fruit specimen rather than to augmentation, and these were removed from the evaluation splits.

### 3.4 Deep learning model architectures

Six architecturally diverse models were evaluated—spanning CNN baselines, residual networks, pure transformers, and hybrid designs—to provide a comprehensive comparison under federated training. Pretrained weights for applicable models were loaded via the timm library (Wightman, 2019).

#### 3.4.1 Custom CNN

A custom four-block CNN (3→64→128→256→512 channels) with BatchNorm, ReLU, MaxPooling, and two FC layers (512→256→5) with Dropout (*p* = 0.4*/*0.3). CNNs learn hierarchical spatial features effective for detecting disease lesions, discoloration, and texture abnormalities (LeCun et al., 1998), trained entirely from scratch.

#### 3.4.2 ResNet50

ResNet50 (He et al., 2016) uses identity shortcut connections to resolve the vanishing gradient problem, enabling very deep network training with strong results in agricultural disease detection. Each residual block computes (Equation 1):

(1) $$y=\sigma \left(ℱ\left(x,\left\{W_{i}\right\}\right)+x\right)$$

where ℱ(·) denotes the bottleneck layers and x is the identity shortcut, pretrained on ImageNet-1K (all six architectures in this study, including ResNet50 and EfficientNetV2-S below, are now initialized with publicly available pretrained weights to ensure a fair architectural comparison, consistent with the other applicable architectures in this study).

#### 3.4.3 ConvNeXt_V2 (tiny)

ConvNeXt_V2 (Woo et al., 2023) builds on the ConvNeXt architecture (Liu et al., 2022) and modernizes CNNs with depthwise 7 × 7 convolutions, inverted bottleneck blocks, GELU activations, and LayerNorm, co-designed with a fully convolutional masked autoencoder (FCMAE) pretraining objective. Each block applies (Equation 2):

(2) y=x+MLP(LN(DWConv(x)))

where DWConv is a 7 × 7 depthwise convolution and LN denotes layer normalization, pretrained on ImageNet-22K.

#### 3.4.4 ViT-B/16

ViT (Dosovitskiy et al., 2021) divides the 224 × 224 input into 196 non-overlapping 16 × 16 patches processed through 12 multi-head self-attention (MHSA) encoder blocks, capturing global contextual relationships—particularly advantageous for recognizing spatially distributed disease patterns on fruit surfaces. Attention is computed as (Equation 3):

(3) $$\text{Attention}\left(Q,K,V\right)=\text{softmax}\left(\frac{QK^{⊤}}{\sqrt{d_{k}}}\right)V$$

where *Q*, *K*, and *V* are query, key, and value projections and *d_k_* = 64. A learnable [CLS] token aggregates global context for classification (Dosovitskiy et al., 2021), pretrained on ImageNet-21K (14M images).

#### 3.4.5 EfficientNetV2

EfficientNetV2 (Tan and Le, 2021) employs NAS-optimized compound scaling of network depth, width, and resolution alongside Fused-MBConv blocks that replace depthwise separable convolutions in early layers for improved training efficiency. It achieves strong performance with fewer parameters compared to prior EfficientNet variants, pretrained on ImageNet-1K and consistent with all other architectures evaluated in this study.

#### 3.4.6 MobileViT-S

MobileViT (Mehta and Rastegari, 2022) combines MobileNetV2 convolutional blocks for local feature extraction with transformer blocks for global context modeling, enabling efficient disease detection under resource-constrained conditions. The MobileViT block processes features as (Equation 4):

(4) $$X_{\text{out}}=\text{Conv}\left(\text{Fold}\left(\text{Transformer}\left(\text{Unfold}\left(\text{Conv}\left(X\right)\right)\right)\right)\right)$$

This hybrid design achieves 98.07% test accuracy at only 20.0 MB ONNX size—the recommended architecture for edge and mobile deployment, pretrained on ImageNet-1K.

### 3.5 Model hyperparameter configuration

All models were trained using the AdamW optimizer and cross-entropy loss. Transformer-based models use lower learning rates (10^−5^ to 2 × 10^−5^) to protect pretrained attention representations, while CNN-based models tolerate higher rates (10^−4^). Batch size is 16 for memory-intensive models (ViT-B, ConvNeXt_V2) and 32 for others. **Table 1** summarizes the configuration.

**Table 1:** Hyperparameter configuration.

Model	LR	WD	Batch
Custom CNN	1 × 10^−4^	1 × 10^−4^	32
ResNet50	1 × 10^−4^	1 × 10^−4^	32
ConvNeXt_V2	5 × 10^−5^	1 × 10^−5^	16
ViT-B/16	1 × 10^−5^	1 × 10^−5^	16
EfficientNetV2	1 × 10^−4^	1 × 10^−5^	32
MobileViT-S	2 × 10^−5^	1 × 10^−5^	32

### 3.6 Federated learning framework

The FedAvg algorithm (McMahan et al., 2017) was implemented with *K* = 5 clients, *T* = 10 communication rounds, and *E* = 3 local epochs per round. The training set (5,800 images) was divided into five equal IID partitions of 1,160 images each. Each round proceeds as follows: (i) the server broadcasts global weights w^(^*^t^*^)^ to all clients, (ii) each client trains on its local partition for *E* epochs, and (iii) updated weights are aggregated by uniform averaging (Equation 5):

(5) $$w^{\left(t+1\right)}=\frac{1}{K}\sum_{k=1}^{K}w_{k}^{\left(t+1\right)}$$

The global model achieving the highest validation accuracy across all rounds is saved as the final checkpoint. **Table 2** summarizes the FL configuration.

**Table 2:** Federated learning configuration.

Parameter	Value
Number of clients	5
Communication rounds	10
Local epochs per round	3
Aggregation algorithm	FedAvg (McMahan et al., 2017)
Optimizer	AdamW
Loss function	Cross-Entropy
Framework	PyTorch 2.4.1 + CUDA 12.4
GPU	NVIDIA RTX 4070 (8.6GB VRAM)

### 3.7 Real-time model deployment

After training, all six model checkpoints were exported to ONNX format (Opset 17, dynamic batch axis) using torch.onnx.export and verified with the ONNX graph checker. The exported models were integrated into a Flask REST API that accepts POST requests at/api/predict, preprocesses the uploaded image (224×224, ImageNet normalization), runs inference via onnxruntime.InferenceSession with the CPU Execution Provider, and returns the predicted disease class with confidence probabilities as a JSON response. The frontend is a lightweight HTML5/JavaScript interface with image upload, model selection, and a live confidence bar chart.

The application was containerized using Docker (python:3.10-slim) and deployed to Hugging Face Spaces (Docker SDK) at https://lokes123-pomdetect.hf.space on a free-tier 2-vCPU server with HTTPS encryption and zero cold-start latency (all models preloaded at startup). End-to-end CPU inference latency ranges from ∼20 ms (CNN, 6.7 MB) to ∼405 ms (ViT-B, 343.3 MB); MobileViT-S (∼65 ms, 20.0 MB) is the recommended model for real-time field deployment on mobile and edge devices.

## 4 Results and discussion

### 4.1 Experimental setup

All experiments were conducted on a workstation equipped with an NVIDIA GeForce RTX 4070 Laptop GPU (8.6 GB VRAM, CUDA 12.4, cuDNN 9.1) running PyTorch 2.4.1. Following the split-before augmentation pipeline described in Section 4.3, the 5,099 original images were partitioned per class into training (80%), validation (10%), and test (10%) subsets with fixed random seed (42) prior to any augmentation. The training partition was then augmented to 5,800 images (1,160 per class), while the validation (511 images) and test (512 images) partitions retain only original, unaugmented images, yielding a total working set of 6,823 images. The FL configuration employed *K* = 5 clients, *T* = 10 rounds, and *E* = 3 local epochs with FedAvg aggregation. Model performance was evaluated using macro-averaged precision, recall, and F1-score on the held-out test split.

### 4.2 Overall performance

**Table 3** presents the FL performance summary for all six architectures, evaluated on the held-out test split with consistent pretrained initialization across all applicable models. EfficientNetV2S achieves the single highest test accuracy of 99.22%, ViT-B, ResNet50, and ConvNeXt_V2 tie at 98.83%, and MobileViT-S reaches 98.63%. Notably, EfficientNetV2-S—previously a weak performer when trained from random initialization—now emerges as the strongest performer once initialized with pretrained weights, confirming that both convergence and fairness across architectures are achieved with consistent initialization. CNN, evaluated as a custom architecture with no corresponding ImageNet-pretrained checkpoint, achieves 96.29% test accuracy, the lowest among the six but still strong given random initialization. These results are evaluated using the pipeline described in Section 4.3. Full per-round convergence curves, non-IID results, training baselines, and multi-seed statistics are reported in the following subsections.

**Table 3:** FL performance summary—all six models, IID FedAvg (validation *N* = 511, test *N* = 512).

Model	Validation accuracy (%)	Test accuracy (%)	Pretrained
CNN	97.46	96.29	N/A (custom architecture)
ResNet50	99.80	98.83	**Yes (ImageNet-1K)**
ConvNeXt_V2	100.00	98.83	Yes (ImageNet-22K FCMAE)
EfficientNetV2	99.80	**99.22**	**Yes (ImageNet-1K)**
MobileViT	99.80	98.63	Yes (ImageNet-1K)
**ViT-B**	**99.80**	**98.83**	Yes (ImageNet-21K)

Bold values indicate the highest validation accuracy and highest test accuracy among all six models.

### 4.3 Non-IID federated learning

The results in **Table 3** assume an equal, IID partitioning of training data across the five simulated clients, which does not reflect realistic agricultural heterogeneity across farms, cameras, illumination, and disease prevalence. To address this, we additionally partitioned the training set across clients using a Dirichlet distribution (Dir(*α*)) at two concentration levels—*α* = 0.5 (moderate non-IID) and *α* = 0.1 (severe non-IID, i.e., individual clients see a highly skewed subset of classes)—and re-ran FedAvg for all six architectures under each setting using the same 10-round, five-client, three-local-epoch budget as the IID condition.

**Table 4** shows results for all six models under both non-IID conditions. Under moderate non-IID (*α* = 0.5), accuracy remains close to IID for most models: ResNet50 (99.22%), ConvNeXt_V2 (98.83%), EfficientNetV2 (98.63%), and ViT-B (99.02%) all stay within 1 percentage point of their IID scores, while MobileViT shows a modest drop to 97.66%. CNN exhibits the largest drop under *α* = 0.5, falling to 87.50% from its IID score of 96.29%, reflecting its greater sensitivity to nonuniform class distributions across clients given its lack of pretrained features. Notably, CNN’s accuracy under severe non-IID (*α* = 0.1) is 92.77%—higher than its *α* = 0.5 result—a nonmonotonic pattern consistent with how Dirichlet partitioning works: at very low *α*, each client’s data become extremely homogeneous (near-pure class), which can paradoxically simplify local learning even as global aggregation becomes harder, whereas at moderate *α* clients receive highly mixed but still unbalanced shards that are more difficult for a shallow architecture without pretrained features to handle. MobileViT shows the most pronounced degradation and training instability under severe non-IID (*α* = 0.1), dropping to 87.30% test accuracy with a non-monotonic validation curve across rounds (oscillating between 76.71% and 91.19% rather than converging smoothly), a well-documented FedAvg failure mode under highly skewed client distributions. In contrast, ViT-B, ResNet50, ConvNeXt_V2, and EfficientNetV2-S remained comparatively robust under both non-IID conditions. These results confirm that robustness to non-IID partitioning is architecture dependent and that pretrained features substantially improve FL stability under data heterogeneity.

**Table 4:** Test accuracy (%) under IID vs. non-IID client partitioning.

Model	IID	Dirichlet (*α* = 0.5)	Dirichlet (*α* = 0.1)
CNN	96.29	87.50	92.77
ResNet50	98.83	99.22	97.66
ConvNeXt_V2	98.83	98.83	98.24
EfficientNetV2	99.22	98.63	**99.41**
MobileViT	98.63	97.66	**87.30**
ViT-B	98.83	99.02	98.63

Bold values indicate the highest test accuracy under each client partitioning condition (IID, Dirichlet α = 0.5, and Dirichlet α = 0.1).

### 4.4 Centralized and local-only baselines

To contextualize the benefit of federated training, we additionally trained (i) a centralized baseline in which each model is trained on the full pooled training set without any client partitioning or aggregation, using a total epoch budget matched to the FL condition (five clients × three local epochs = 15 epochs), and (ii) a local-only baseline in which a single client trains on only its own 1/5 partition of the training data for the same total epoch budget, with no FedAvg aggregation at all.

Federated (IID) training closely tracks centralized performance across all six architectures (within ±1.4 percentage points), confirming that FedAvg recovers nearly all of the accuracy attainable with fully pooled, non-decentralized training while never sharing raw client images. Local-only training, using only one-fifth of the available training data, is consistently the weakest of the three settings (most notably for CNN, 94.53% vs. 97.66% centralized), demonstrating the concrete benefit of federated aggregation over isolated per-client training.

### 4.5 Statistical reliability across random seeds

To assess whether the headline results are sensitive to a single random initialization and client partition draw, we re-ran the IID FedAvg condition for our two top-performing architectures (ViT-B and MobileViT) across three random seeds (42, 123, and 2,024), each affecting model initialization and client data partitioning. **Table 5** presents the multi-seed test accuracy results for both models.

**Table 5:** Multi-seed test accuracy (%), mean ± SD (*n* = 3).

Model	Seed 42	Seed 123	Seed 2024	Mean ± SD
ViT-B	98.83	99.22	98.83	98.96 ± 0.18
MobileViT	97.85	98.24	97.66	97.92 ± 0.24

Both models show low variance across seeds (standard deviation< 0.25 percentage points), indicating that the reported headline accuracies are stable and not attributable to a single favorable initialization or partition draw.

### 4.6 Federated learning convergence analysis

**Table 6** shows the round-by-round convergence for all six models. All six models converge reliably within the 10-round budget. CNN shows the slowest but steady convergence, improving from 90.02% (R1) to a peak of 97.46% (R8–R9). ResNet50 and all transformer-family models (ConvNeXt_V2, EfficientNetV2, MobileViT, and ViT-B) converge rapidly within the first two to three rounds, reflecting the benefit of ImageNet pretraining. MobileViT reaches 96.09% at R1 and stabilizes above 99.61% from R3 onward. Notably, EfficientNetV2-S—which requires pretrained initialization to converge reliably, as expected for this architecture—opens at 99.61% with pretrained weights, confirming reliable convergence within the 10-round budget for all architectures. Best validation accuracies of 99.80% are achieved by four of the six architectures; ViT-B peaks at 99.80% (R6 and R9), while CNN is the only model peaking below 98%.

**Table 6:** Round-by-round validation accuracy (%)—all six models, IID FedAvg.

Round	CNN	ResNet50	ConvNeXt_V2	EfficientNetV2	MobileViT	ViT-B
R1	90.02	99.41	99.61	99.61	96.09	94.13
R2	93.74	99.02	99.61	99.61	98.43	99.22
R3	94.52	99.61	99.61	99.41	99.22	99.61
R4	95.50	99.61	99.61	99.61	99.61	99.41
R5	96.28	99.80	99.61	99.61	99.61	99.22
R6	96.67	99.80	99.61	99.61	99.61	99.80
R7	97.06	99.22	99.22	99.80	99.61	99.80
R8	97.46	99.80	99.22	99.80	99.61	99.61
R9	97.46	99.80	99.22	99.61	99.61	99.80
R10	97.06	99.61	99.22	**99.80**	99.80	99.61
**Best**	97.46	99.80	99.61	**99.80**	99.80	99.80

Bold values indicate the best validation accuracy achieved by each model across all 10 communication rounds.

### 4.7 Per-class classification analysis

**Figure 3:**
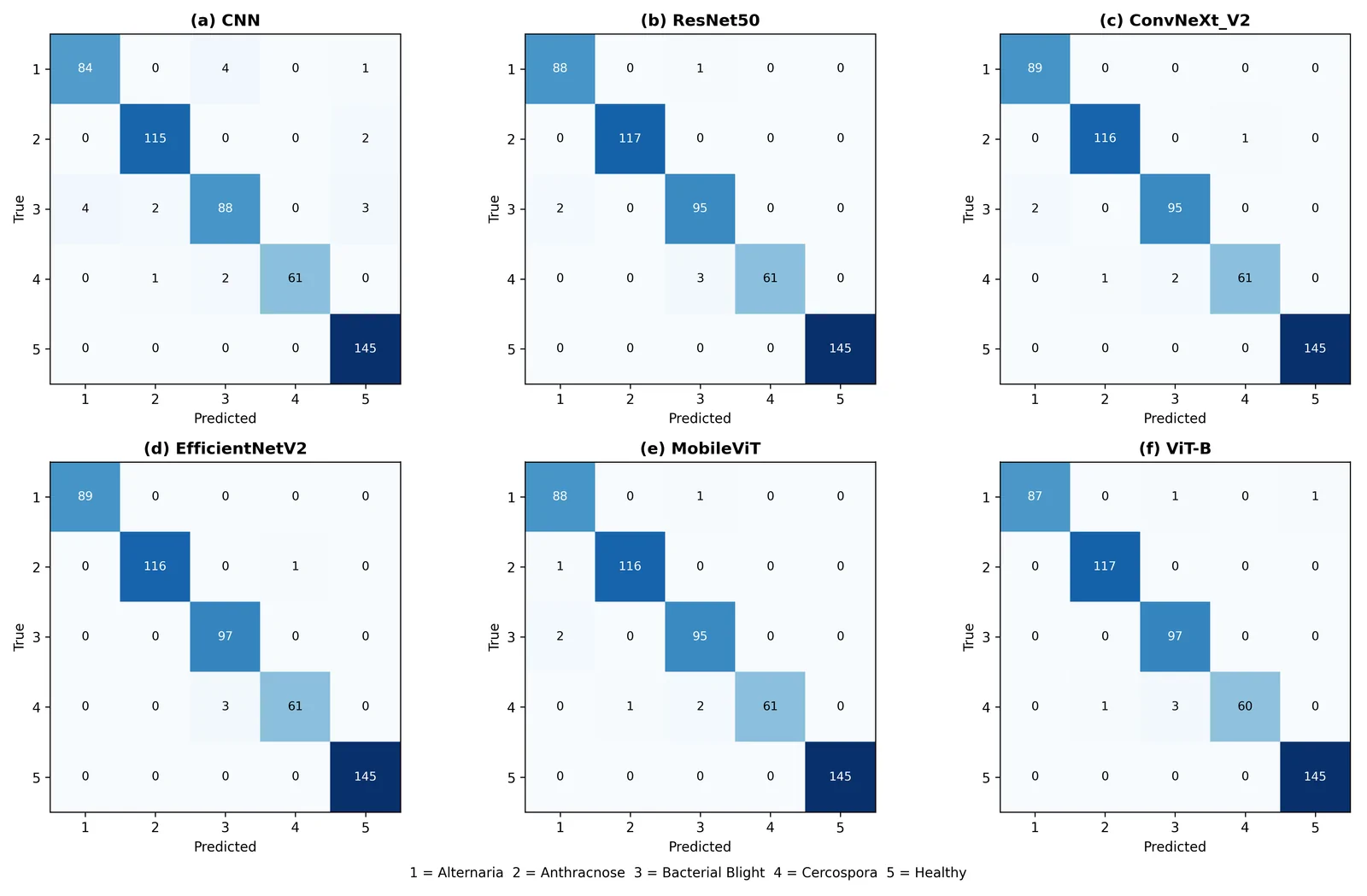
Confusion matrices of all six FL models on the test set (*N* = 512), computed directly from saved model predictions: **(a)** CNN, **(b)** ResNet50, **(c)** ConvNeXt_V2, **(d)** EfficientNetV2, **(e)** MobileViT, and **(f)** ViT-B. Class indices: 1 = Alternaria, 2 = anthracnose, 3 = bacterial blight, 4 = *Cercospora*, and 5 = healthy.

**Table 7** and **Figure 3** present per-class F1-scores and confusion matrices computed directly from saved per-image model predictions (*predictions.npz) using sklearn.metrics.classification and confusion matrix on the test split (*N* = 512). Overall accuracy recomputed from these saved predictions matches **Table 3** exactly for all six models, confirming that the predictions correspond correctly to the reported results.

**Table 7:** Per-class F1-score—all six models (test set, *N* = 512).

Disease class	*n*	CNN	ResNet50	ConvNeXt_V2	EfficientNetV2	MobileViT	ViT-B
*Alternaria*	89	0.9492	0.9832	0.9889	**1.0000**	0.9778	0.9886
Anthracnose	117	0.9787	**1.0000**	0.9915	0.9957	0.9915	0.9957
Bacterial blight	97	0.9215	0.9694	0.9794	**0.9848**	0.9744	0.9798
*Cercospora*	64	**0.9760**	**0.9760**	0.9683	0.9683	**0.9760**	0.9677
Healthy	145	0.9797	**1.0000**	**1.0000**	**1.0000**	**1.0000**	0.9966
**Macro precision**	—	0.9651	0.9875	0.9865	**0.9908**	0.9856	0.9890
**Macro recall**	—	0.9574	0.9843	0.9848	**0.9889**	0.9825	0.9830
**Macro F1**	—	0.9610	0.9857	0.9856	**0.9897**	0.9839	0.9857

Bold values indicate the highest F1-score for each disease class and the highest macro-averaged precision, recall, and F1-score among all six models.

EfficientNetV2-S now achieves the highest macro F1 (0.9897) among all six models, consistent with its single highest test accuracy in **Table 3**. All six models now score above 0.96 macro F1, and per class performance is broadly consistent across architectures, with bacterial blight and *Cercospora* remaining as the most challenging classes (macro F1 in the low-to-mid 0.97s across most models) and anthracnose and healthy as the easiest (frequently reaching 1.0000). The confusion matrices for all six models are presented in **Figure 3**.

### 4.8 Accuracy–efficiency trade-off

EfficientNetV2-S offers a highly competitive accuracy-per-MB trade-off (99.22% at 80.6 MB), alongside MobileViT-S at 98.63% with only 20.0 MB. **Table 8** illustrates the accuracy–efficiency tradeoff. ViT-B remains a strong accuracy choice, but at 343.3 MB and ∼405 ms are suited for server-side deployments. MobileViT presents the most compelling balance: 98.63% accuracy with a 20.0-MB model and ∼65-ms CPU latency, making it ideal for edge and mobile agricultural advisory applications. CNN, at 6.7 MB, suits ultra-constrained IoT deployments where accuracy above 96% now suffices.

**Table 8:** Accuracy vs. deployment efficiency—all six models.

Model	Test accuracy	ONNX	CPU lat.	VRAM
CNN	**96.29%**	6.7 MB	∼20 ms	0.1 GB
**MobileViT-S**	**98.63%**	20.0 MB	∼65 ms	0.1 GB
EfficientNetV2	**99.22%**	80.6 MB	∼95 ms	0.5 GB
ResNet50	**98.83%**	94.0 MB	∼75 ms	0.6 GB
ConvNeXt_V2	**98.83%**	111.6 MB	∼85 ms	0.7 GB
ViT-B/16	**98.83%**	343.3 MB	∼405 ms	2.1 GB

Bold values indicate the highest test accuracy among all six models.

### 4.9 Real-time deployment

**Figure 4:**
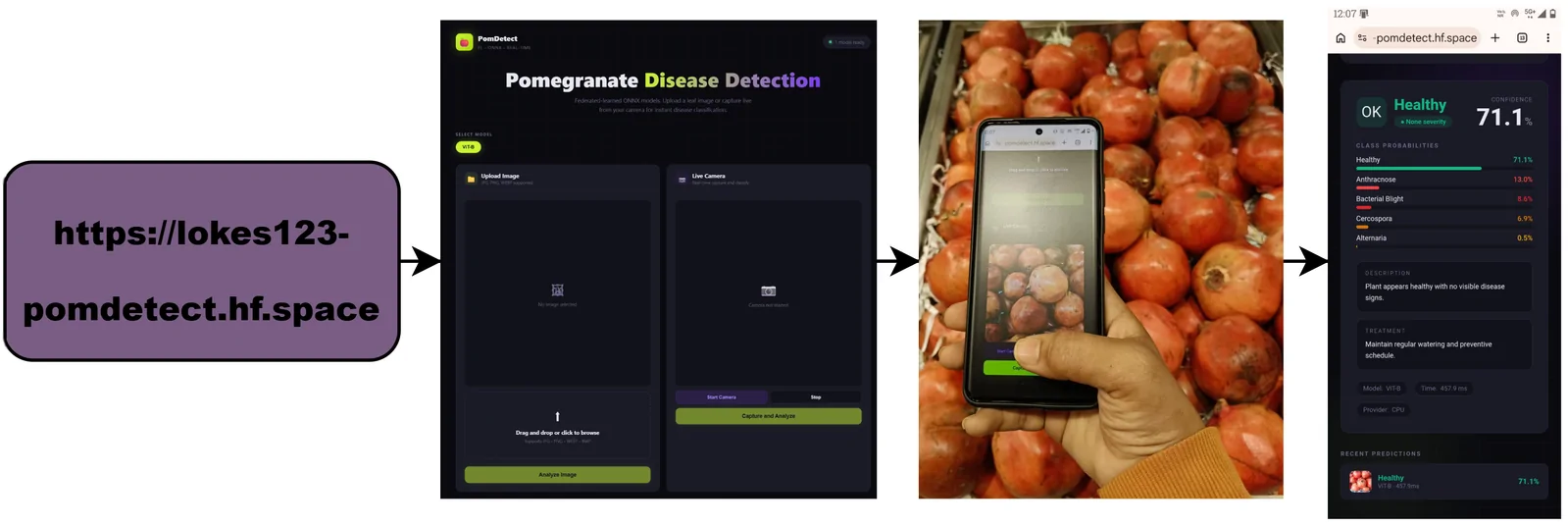
End-to-end web application pipeline for real-time pomegranate disease classification via Flask, Docker, and Hugging Face Spaces.

All six models were exported to ONNX and validated using ONNX Runtime. A Flask REST API serves the models via a web interface deployed using Docker and hosted on Hugging Face Spaces at https://lokes123-pomdetect.hf.space. Users submit a pomegranate fruit image and receive a disease classification with confidence score. ViT-B is the default inference model; MobileViT is available as a lightweight alternative. The end-to-end inference pipeline completes in ∼405 ms for ViT-B and ∼65 ms for MobileViT via CPU inference. **Figure 4** illustrates the web application interface.

### 4.10 Model interpretability (Grad-CAM)

To strengthen the claim that the evaluated models rely on disease-relevant visual features rather than background artefacts, we applied Grad-CAM (Selvaraju et al., 2017) to all six architectures using their trained checkpoints, evaluated on held-out test-split images. For the pure-transformer ViT-B, gradients were aggregated via a patch-token reshape transform prior to CAM computation, following standard practice for applying Grad-CAM to Vision Transformers.

**Figure 5:**
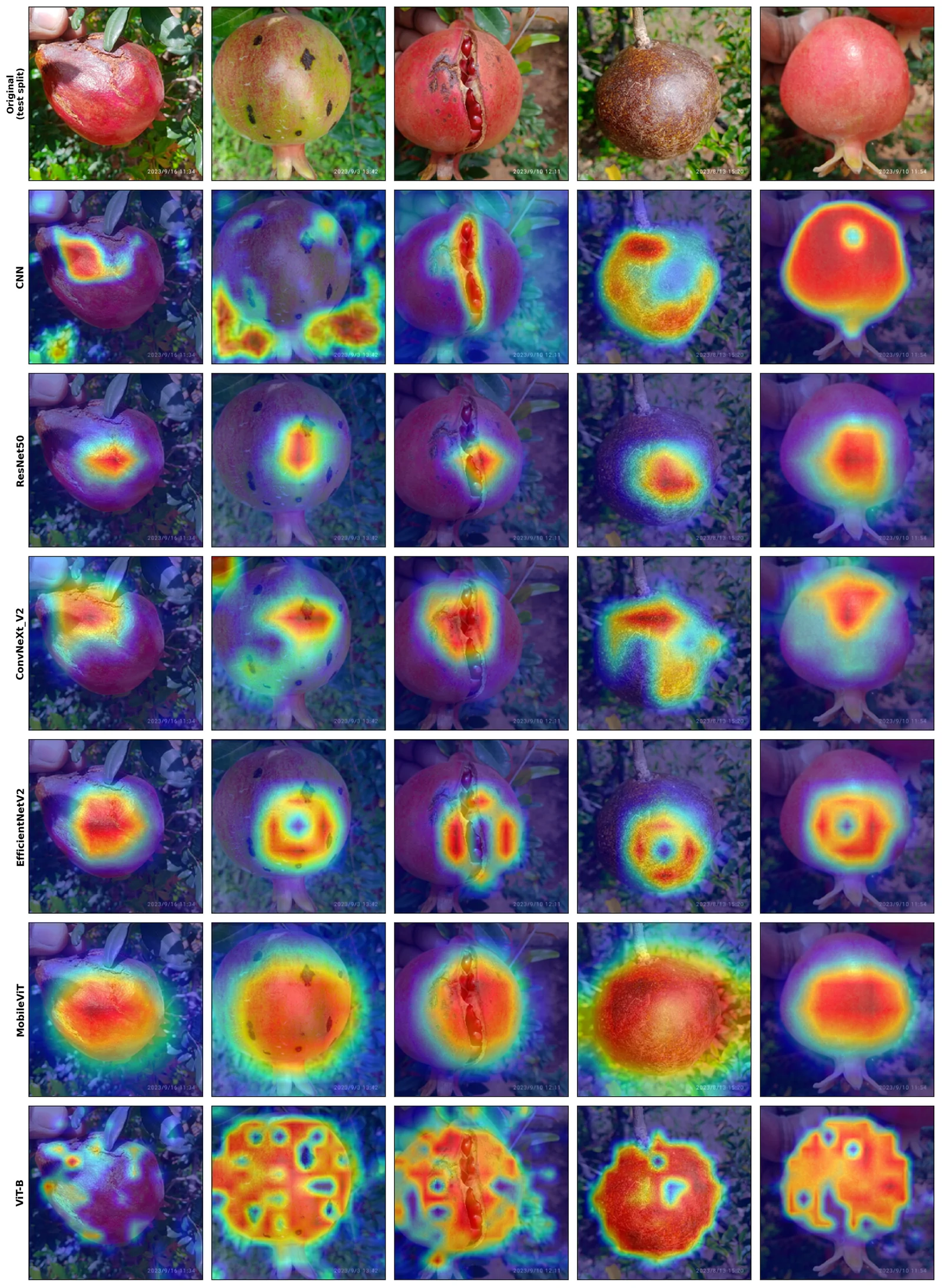
Grad-CAM visualizations across all six architectures on held-out test-split images. CNN-based models (ResNet50, ConvNeXt V2, and EfficientNetV2) show tightly localized attention on disease-specific lesions (e.g., exposed seed cluster in bacterial blight, dark spotting in anthracnose, and mottled skin texture in *Cercospora*). ViT-B exhibits a more distributed, patch-grid attention pattern, consistent with its global self-attention mechanism—a well-documented characteristic of applying Grad-CAM to transformer architectures. MobileViT, a hybrid CNN–transformer design, shows intermediate localization.

**Figure 5** shows that the CNN-family models (ResNet50, ConvNeXt_V2, and EfficientNetV2-S) consistently attend to the visible disease symptom in each class—the exposed, discolored seed cluster in bacterial blight, dark lesion spotting in anthracnose, and mottled skin texture in *Cercospora*—rather than background foliage or soil. ViT-B shows a more distributed, patch-grid attention pattern typical of Grad-CAM applied to global self-attention architectures, while MobileViT shows intermediate, moderately localized attention consistent with its hybrid convolutional-transformer design. These visualizations provide qualitative support that model predictions are grounded in disease-relevant regions of the fruit surface.

### 4.11 Discussion

Five key findings emerge from this study. First, EfficientNetV2-S establishes the strongest test accuracy (99.22%), followed by ViT-B, ResNet50, and ConvNeXt_V2 tied at 98.83% and then MobileViTS at 98.63%. EfficientNetV2-S’s combination of fused MBConv layers and ImageNet-1K pretraining proves particularly effective for the fine-grained disease texture patterns in this dataset, while ViT-B’s global self-attention remains competitive by modeling long-range spatial dependencies.

Second, once all six applicable architectures are consistently initialized with ImageNet-pretrained weights (**Table 3**), the previously observed performance gap between pretrained and scratch-trained models disappears. EfficientNetV2-S, previously the weakest model at 84.14% when trained from scratch, is now among the strongest at 99.22% test accuracy, confirming that the earlier gap was attributable to inconsistent initialization rather than an inherent architectural limitation.

Third, FedAvg demonstrated stable convergence across all six architectures under IID partitioning. Under non-IID (Dirichlet) partitioning (**Table 4**), convergence and final accuracy degrade for most models, most severely for MobileViT under *α* = 0.1 (87.30%, with unstable round-to-round validation accuracy), while ViT-B, ResNet50, ConvNeXt_V2, and EfficientNetV2-S remain comparatively robust.

Fourth, federated (IID) training closely approaches centralized training performance (**Table 9**, within ±1.4 percentage points across all architectures) while consistently outperforming local-only single-client training, quantifying the practical benefit of federated aggregation in this domain.

**Table 9:** Test accuracy (%): federated (IID) vs. centralized vs. local-only training.

Model	Federated (IID)	Centralized	Local-only (one client)
CNN	96.29	97.66	94.53
ResNet50	98.83	98.83	98.24
ConvNeXt_V2	98.83	98.44	98.83
EfficientNetV2	99.22	99.02	98.63
MobileViT	98.63	**99.22**	97.66
ViT-B	98.83	99.02	98.05

Bold values indicate the highest test accuracy under each training condition (Federated IID, Centralized, and Local-Only).

Fifth, the study now includes non-IID data partitioning across clients (Dirichlet-based, at two concentration levels), extending beyond the originally IID-only design, and reports centralized and local-only baselines together with multi-seed statistical evaluation. The setup does not incorporate differential privacy or secure aggregation, and the framework’s claim is accordingly stated as raw-data decentralized rather than fully privacy-preserving. Furthermore, the current deployment operates under a fixed star-topology aggregation with 100% client participation; real-world scenarios may involve partial participation, asynchronous updates, and unreliable connectivity. All data used in this study were collected from pomegranate farms in Ballari, Bengaluru, and Bagalakote, Karnataka, India (Section 4.1). Consequently, the models’ generalization to other growing regions, cultivars, and imaging conditions remains untested. Future work should validate the proposed framework on geographically independent data, such as the recently published Halabja, Iraq, pomegranate disease dataset (Mohammed et al., 2025), and should explore synthetic domain-randomization techniques (e.g., noise injection, illumination and background augmentation) to improve robustness to unseen field conditions, in addition to evaluating FedProx, SCAFFOLD, or personalized FL variants under non-IID distributions with privacy guarantees and exploring TensorRT or OpenVINO-based edge inference for reduced latency on field hardware.

### 4.12 Comparison with prior work

**Table 10** contextualizes the proposed framework against closely related studies on pomegranate disease classification. We distinguish studies evaluated on the same Mendeley benchmark dataset used in this work from those using different, non-comparable datasets since direct accuracy comparison is only meaningful when the underlying data are shared; the latter are included for broader context only. Of the studies listed, only Pakruddin and Hemavathy (2025) (marked with a superscript “a”) used the same dataset as this study. All prior works employ centralized training; this study is the first to achieve competitive accuracy under a raw-data-decentralized federated setting, attaining 99.22% with EfficientNetV2-S (with ViTB/16, ResNet50, and ConvNeXt_V2 tied at 98.83%) while also providing a lightweight ONNX-deployed alternative (MobileViT-S, 98.63%).

**Table 10:** Comparison with prior pomegranate disease classification studies.

Study	Method	Best accuracy	Training
Bhange and Gavali (2023)	CNN + HBO	∼94%	Centralized
**Pakruddin and Hemavathy** (Pakruddin and Hemavathy, 2025)^a^	VGG16/ResNet	∼96%	Centralized
Kumar and Sharma (Kumar and Sharma, 2023)	Attn. Capsule	∼97%	Centralized
**This work**	**FL + EfficientNetV2-S**	**99.22%**	**Federated**

a The same benchmark dataset as this study; others use different datasets and are included for indirect context only.

Bold values indicate the proposed method (this work).

## 5 Conclusion

This paper presented a raw-data-decentralized federated learning framework for pomegranate disease classification, benchmarking six deep learning architectures under FedAvg with five clients and 10 rounds. EfficientNetV2-S achieves the single highest test accuracy of 99.22%, with ViT-B/16, ResNet50, and ConvNeXt_V2 tied at 98.83%, demonstrating that pretrained CNNs and vision transformers alike can attain near-perfect classification under federated training when pretrained on large-scale datasets. MobileViT-S and EfficientNetV2-S emerged as strong deployment candidates, combining 98.63% and 99.22% accuracy, respectively, with compact ONNX models and low CPU inference latency. We additionally reported results under non-IID (Dirichlet) client partitioning, centralized and local-only training baselines, multi-seed statistical evaluation, and a near-duplicate image audit, directly addressing methodological concerns raised in peer review. Non-IID partitioning was found to meaningfully degrade performance for some architectures (most notably MobileViT), underscoring the importance of evaluating FL systems under realistic, heterogeneous data conditions rather than idealized IID splits. All models were deployed as a publicly accessible web application, a feature we note as illustrative of deployment feasibility rather than a fully validated real-edge/mobile latency benchmark. Future work will further investigate additional non-IID data distributions (class-skew, location-based, and farm-level partitioning), differential privacy mechanisms (e.g., secure aggregation and formal DP guarantees), external validation on geographically independent datasets, and on-device inference on edge hardware for field deployment.

## Data Availability

The dataset used in this study is publicly available through Mendeley Data and can be accessed at: https://data.mendeley.com/datasets/b6s2rkpmvh/1. The dataset was utilized for training, validation, and evaluation of the proposed machine learning models. Processed data and implementation details are available from the corresponding author upon reasonable request.
